# Tobacco Use, Insulin Resistance, and Risk of Type 2 Diabetes: Results from the Multi-Ethnic Study of Atherosclerosis

**DOI:** 10.1371/journal.pone.0157592

**Published:** 2016-06-20

**Authors:** Rachel J. Keith, Mahmoud Al Rifai, Christopher Carruba, Natasha De Jarnett, John W. McEvoy, Aruni Bhatnagar, Michael J. Blaha, Andrew P. Defilippis

**Affiliations:** 1 Diabetes and Obesity Center, University of Louisville School of Medicine, Louisville, Kentucky, United States of America; 2 Division of Cardiology, Department of Medicine, University of Louisville School of Medicine, Louisville, Kentucky, United States of America; 3 American Heart Association—Tobacco Regulatory and Addiction Center, Louisville, Kentucky, United States of America; 4 Ciccarone Center for the Prevention of Heart Disease, John Hopkins Medical, Baltimore, Maryland, United States of America; 5 Department of Medicine, University of Colorado, Aurora, Colorado, United States of America; Beijing Key Laboratory of Diabetes Prevention and Research, CHINA

## Abstract

**Introduction:**

Tobacco use is associated with insulin resistance and incident diabetes. Given the racial/ethnic differences in smoking patterns and incident type 2 diabetes our objective was to evaluate the association between tobacco use and insulin resistance (IR) as well as incident type 2 diabetes mellitus in a contemporary multiethnic cohort.

**Methods and Results:**

We studied 5,931 Multi- Ethnic Study of Atherosclerosis (MESA) participants who at baseline were free of type 2 diabetes (fasting glucose ≥7.0 mmol/l (126 mg/dl) and/or use of insulin or oral hypoglycemic medications) categorized by self-reported tobacco status and reclassified by urinary cotinine (available in 58% of participants) as never, current or former tobacco users. The association between tobacco use, IR (fasting plasma glucose, insulin, and the homeostatic model assessment of insulin resistance (HOMA-IR)) and incident diabetes over 10 years was evaluated using multivariable linear regression and Cox proportional hazards models, respectively. Mean age of the participants was 62 (±10) years, 46% were male, 41% Caucasian, 12% Chinese, 26% African American and 21% Hispanic/Latino. IR biomarkers did not significantly differ between current, former, and never cigarette users (P >0.10) but showed limited unadjusted differences for users of cigar, pipe and smokeless tobacco (All P <0.05). Fully adjusted models showed no association between dose or intensity of tobacco exposure and any index of IR. When stratified into participants that quit smoking vs. those who continued smoking during the 10-year study there was no difference in serum glucose levels or frequency of diabetes. In fully adjusted models, there was no significant difference in diabetes risk between former or current cigarette smokers compared to never smokers [HR (95% CI) 1.02 (0.77,1.37) and 0.81 (0.52,1.26) respectively].

**Conclusion:**

In a contemporary multi-ethnic cohort, there was no independent association between tobacco use and IR or incident type 2 diabetes. The role smoking plays in causing diabetes may be more complicated than originally thought and warrants more in-depth large contemporary multi-ethnic studies.

## Introduction

Tobacco smoke and tobacco products, such as cigars, pipes, and smokeless tobacco, contain many harmful and potentially harmful constituents (HPHC) [1, 2] that affect different organ systems and physiological processes in a tissue-specific manner. Smoking is reported to be a risk factor for type 2 diabetes mellitus [3, 4]. Furthermore, the most recent Surgeon General’s report concluded that smoking is a cause of type 2 diabetes and that the risk of developing type 2 diabetes increases with the cumulative number of cigarettes smoked [5] and this idea has been supported by several meta-analysis as well [6, 7]. In fact, compared with non-smokers, current smokers have been reported to have a 30–40% increased risk of type 2 diabetes [5]. Less is currently understood about other forms of tobacco relationship with type 2 diabetes, but there are some reported links to an increased risk for type 2 diabetes [8, 9]. Multiple mechanisms of tobacco exposure’s causation of diabetes have been proposed, including that tobacco exposure directly causes insulin resistance [10, 11].

The relationship between tobacco use and insulin resistance may be confounded by multiple associated variables. For example, Insulin resistance is often associated with an increase body habitus and increased BMI is a known risk factor for diabetes [12]. Smoking has also been linked to an overall lower BMI than that seen in non-smokers [13] however, smoking has be linked to increased central adiposity [11]. The increase in central adiposity may contribute to insulin resistance and diabetes proposed in smokers [11]. Moreover, there is evidence of different patterns of insulin resistance based on race [14] and gender [15]. Furthermore, smoking trends differ between race/ethnicity and genders, which may create different exposure patterns based on race/ethnicity or gender leading to different patterns of IR or incident diabetes. Of note the Hispanic/Latino populations tend to have low rates of smoking [16] while African American populations tend to have greater use of cigarettes that contain menthol [17].

Temporal trends in tobacco use may also influence the association of IR and incident diabetes. Over the decades the changing composition of cigarettes including the introduction of filters on cigarettes in the mid to late 1950’s which did not gain popularity until the 1980’s [18] and introduction of low tar cigarettes in the 1980’s [19]. Moreover, curing of tobacco and flavoring of tobacco affects the amount and type of HPHC in the product [20, 21], therefore different tobacco use patterns lead to varying exposure to these HPHC’s. There is little research into how these different HPHC’s impact IR and incident diabetes between races.

Indeed, there is a body of evidence showing no relationship between tobacco use and IR or incident diabetes. Current research demonstrates heterogeneity in the association between smoking and glycated hemoglobin (HbA1c) levels, fasting blood glucose levels and 2 hour- post-challenge glycaemia (2H-PG) [22–25]. Furthermore it has been suggested the effect of smoking is not seen in lean individuals, instead only in obese men [26]. Two meta-analysis used to support the causal relationship between tobacco use and diabetes show considerable heterogeneity in the evidence used to complete the study [5, 6]. Indeed, extant meta-analyses used prior studies that often examined the association of smoking and type 2 diabetes in participants of similar race and gender, typically Caucasian males [5, 7].

In light of the heterogeneity of evidence available we aimed to identify whether the association between tobacco use IR and type 2 diabetes suggested in previous studies is also evident in large well characterized multi-ethnic cohort with multiple validated markers of insulin resistance (IR). Moreover, we sought to identify if there were markers of IR sensitive to smoking that may help identify a group of tobacco product users who are at an increased risk of type 2 diabetes. Identifying IR and incident diabetes risk in these in a contemporary multi-ethnic population seemed important as tobacco exposure patterns and metabolism of tobacco differs by race [27–30], and gender, [25, 28, 31]. We therefore examined the relationship between tobacco exposure, cross-sectional insulin resistance (IR) at baseline, and prospective incident type 2 diabetes over the course of 10 years in a contemporary multi-ethnic cohort.

## Methods

The Multi-Ethnic Study of Atherosclerosis (MESA) is a prospective epidemiological study of the prevalence, risk factors, and progression of subclinical CVD in a multi-ethnic cohort. The study design and methods have been previously published [32]. Briefly, 6,814 participants aged 45–84 who identified themselves as Caucasian, African-American, Hispanic/Latino, or Chinese were recruited from 6 U.S. communities (Baltimore City and Baltimore County, Maryland; Chicago, Illinois; Forsyth County, North Carolina; Los Angeles County, California; New York, New York and St. Paul, Minnesota) from 2000–2002. Participants were free of clinical CVD at enrollment. The protocols were approved by the institutional review boards of all collaborating institutions including John Hopkins University, Northwestern University, Wake Forest School of Medicine, University of California Los Angeles, Columbia University, University of Minnesota Clinical and Translational Science Institute, and by the National Heart, Lung, and Blood Institute. All participants provided written informed consent.

### Exclusion Criteria

Criteria for exclusion included baseline type 2 diabetes [fasting glucose ≥7.0 mmol/l (126 mg/dl)] or previous medical diagnosis and/or use of insulin and/or oral hypoglycemic medications at baseline (n = 688, 10%). Prevalence of diabetes at baseline was 13%. Of note, there was no significant difference in the proportion of smokers excluded due to type 2 diabetes at baseline and those who were included in our study (results not shown). Participants were also excluded if they were missing tobacco exposure status (n = 35, 0.5%), duration of tobacco exposure (n = 129, 1.9%), and baseline fasting glucose or insulin levels (n = 31, 0.5%). The final sample size consisted of 5,931 participants.

### Tobacco Exposure

Tobacco use included cigarettes, cigars, pipes, and smokeless tobacco defined as chewing tobacco and/or snuff. Subjects were classified as never (less than 100 lifetime cigarettes or less than 20 cigars, pipefuls, or smokeless tobacco uses), former (no use in past 30 days but more than 100 lifetime cigarettes or 20 cigars, pipefuls, or smokeless tobacco uses), or current users of tobacco products (used tobacco in last 30 days and more than 100 lifetime cigarettes or more than 20 cigars, pipefuls, or smokeless tobacco uses). Self-reported never smokers at baseline who reported being former smokers at visit 2 (n = 143) were reclassified as baseline former smokers. Baseline urinary cotinine, a product of nicotine metabolism that reflects tobacco exposure levels [33], was measured in 3,943 total participants (about half the cohort). 69 subjects (12%) who identified themselves as never or former smokers and had urinary cotinine levels greater than 500 ng/mL were reclassified as being current smokers. For former and current users, dose of exposure was defined in pack years: calculated as number of packs per day of cigarettes smoked multiplied by the number of years of smoking. Dose of pipe, cigars, and smokeless tobacco use was defined as number used per day multiplied by the number of years. Intensity of tobacco exposure was defined as the average number of cigarettes, cigars, pipes, or smokeless tobacco per day.

### Outcomes

Cross-sectional IR was assessed by fasting plasma glucose levels, insulin levels, and HOMA-IR. Fasting blood glucose in serum was measured by the glucose oxidase method using the Vitros 950 analyzer (Johnson & Johnson Clinical Diagnostics, Rochester, New York). Fasting serum insulin was measured by the Linco Human Insulin Specific Radioimmunoassay kit (Linco Research, Inc., St. Charles, Missouri) [34]. HOMA-IR was calculated using the equation: glucose (mg/dL) x insulin (mU/L) / 405. Prediabetes was defined according to the American Diabetes Association as fasting glucose ≥100 & <126 mg/dL. Incident type 2 diabetes was accessed prospectively over a median 10 years of follow-up and was defined as a fasting glucose ≥7.0 mmol/l (126 mg/dl) and/or use of insulin or oral hypoglycemic medications.

### Measurement of Covariates

Information on demographics, medical history, socioeconomic status, diet and physical activity was collected using questionnaires as previously described [32]. Body mass index (BMI) was calculated as weight in kilograms divided by height in meters squared. Systolic and diastolic blood pressure (SBP, DBP) were measured three times using an automated sphygmomanometer and the mean of the last two measurements was used. Triglycerides, total and high-density lipoprotein cholesterol (HDL-C) were measured at a central laboratory (Fairview-University Medical Center, Minneapolis, MN) after a 12-hour fast. Low-density lipoprotein cholesterol (LDL-C) was calculated using the Friedewald equation. High-sensitivity C-reactive protein (hsCRP) was measured using a particle-enhanced immunonepholometric assay on the BNII nephelometer (Dade-Behring, Inc., Deerfield, IL) at the University of Vermont, Burlington, Vermont.

### Statistical Analysis

Baseline characteristics of the population by cigarette smoking status were reported as means ± standard deviation for normally distributed variables and medians (interquartile range) for skewed variables. Differences were tested using ANOVA, equality-of-medians test and chi-squared test. For non-cigarette tobacco exposure, all analyses were restricted to those not currently using cigarettes (n = 5123) in order to minimize confounding due to concurrent cigarette smoking. Linear regression was used to assess for a possible dose-dependent relationship between dose and intensity of tobacco exposure and levels of IR biomarkers. Cox proportional hazards models were used to study the association between tobacco exposure and incident type 2 diabetes. The proportionality assumption was tested using graphic methods. Models were adjusted for age, gender, race/ethnicity, BMI, income, SBP, DBP, anti-hypertensive medication use, HDL-C, LDL-C, lipid-lowering medication use, physical activity, healthy diet, alcohol use and hsCRP.

To further explore and strengthen any potential cause-and-effect relationships between smoking, insulin resistance, and diabetes, we performed several longitudinal data analyses using multiple linear regression adjusting for the same covariates and also including change in insulin resistance and change in smoking exposure. Only serum glucose was available at each of the 5 MESA visits and therefore we could not model change in insulin or HOMA-IR. We accounted for change in smoking status using 3 approaches. In the first approach we restricted our analyses to individuals who maintained their smoking status at all MESA visits. In the second we analyzed individuals who quit smoking cigarettes at visit 2 and maintained their status as former smokers at each of the subsequent MESA visits. In the third approach, we adjusted for time-sensitive terms for quitting cigarette smoking at visits 2, 3, 4, or 5.

As sensitivity analyses, we did not adjust for hsCRP as it may mediate the association between tobacco use and IR/incident diabetes. We also studied the association between tobacco exposure status and pre-diabetes defined as fasting serum glucose between 100 and 126 mg/dL (S1 Data).

All analyses were performed using STATA (version 13) and a p-value of <0.05 was considered statistically significant (two-sided).

## Results

Mean age of the participants was 62 (±10) years, 46% were male, 41% were Caucasian, 12% Chinese, 26% African American, and 21% Hispanic/Latino. Current cigarette smokers were younger compared to never and former smokers (p-values <0.001). Never smokers were more likely to be female while former smokers were more likely to be male. Compared with never smokers, current and former users were more likely to be African American (36% and 27% vs. 23%) and less likely to be Chinese (6% and 7% vs. 18%). Compared with never and former smokers, current smokers had lower SBP, less use of anti-hypertensive medications, lower HDL-C, less use of lipid lowering medications, and higher hsCRP (all p<0.05) (Table 1).

**Table 1:** Baseline characteristics of the population by cigarette smoking status.

	Overall	Never	Former	Current	p-value
	(N = 5,931)	(n = 2,874)	(n = 2,249)	(n = 808)	
**Age, years**	62±10	62±11	63±10	58±9	<0.001
**Gender**					<0.001
Female	54	64	42	49	
Male	46	36	58	51	
**Race/ethnicity**					<0.001
Caucasian	41	36	48	36	
Chinese	12	18	7	6	
African American	26	23	27	36	
Hispanic/Latino	21	23	18	22	
**BMI, kg/m2**	28.0±5.3	27.8±5.4	28.4±5.3	27.9±5.3	<0.001
**Alcohol use**					<0.001
Never	20	34	7	9	
Former	23	18	29	23	
Current	57	48	64	68	
**SBP, mmHg**	126±21	126±22	127±21	123±21	<0.001
**DBP, mmHg**	72±10	71±10	73±10	72±11	0.001
**Anti-HTN med use**	34	34	36	27	<0.001
**HDL, mg/dL**	52 ±15	52±15	52±15	47±14	<0.001
**LDL, mg/dL**	118±31	119±31	118 ±31	118±32	0.41
**Lipid-lowering medication use**	15	14	17	11	<0.001
**hsCRP, mg/L**	1.8 (3.4)	1.7 (3.2)	1.8 (3.2)	2.5 (3.9)	<0.001
**Pack-years**	0 (15)	0 (0)	12 (25)	20 (28)	<0.001
**Glucose, mg/dL**	92.0±20.0	91.8±20.3	92.7±20.2	91.0±18.9	0.91*
**Insulin, mU/L**	8.0 (5.8)	8.2 (5.8)	8.0 (6.0)	7.8 (5.8)	0.12*
**HOMA-IR, %**	1.8 (1.5)	1.8 (1.5)	1.8 (1.6)	1.7 (1.4)	0.21*

Continuous variables are expressed as means ± standard deviation if normally distributed and as medians (interquartile range) if skewed

Categorical variables are expressed as percentages

p-value refers to differences in baseline characteristics by categories of cigarette smoking status which was calculated using ANOVA or the Kruskal Wallis test for continuous variables and Chi-square test for categorical variables.

* refers to differences in unadjusted levels of IR biomarkers by categories of cigarette status which was calculated using a test for trend

### IR and Type of Tobacco Exposure

Among never, current, and former cigarette smokers, there was no significant difference in crude levels of glucose, insulin, or HOMA-IR (p>0.05) (Table 1). Former cigar users had higher levels of all IR biomarkers compared with never and current users (all p<0.05) (Table 2). Current pipe users had higher levels of insulin and HOMA-IR in comparison with former and never smokers (all p<0.05) (Table 2). Current smokeless tobacco users had higher levels of all IR biomarkers compared with never and formers users (all p<0.05) (Table 2). After adjustment for age, gender, race/ethnicity, BMI, income, SBP, DBP, anti-hypertensive medication, HDL, LDL, lipid-lowering medication, and alcohol use former cigar retained higher levels of glucose and HOMA-IR, former pipe users retained higher levels of glucose, while current smokeless tobacco users retained higher levels of all IR biomarkers (all p<0.05) (Table 3).

**Table 2:** Duration of exposure and levels of insulin resistance biomarkers in non-cigarette tobacco.

	Never	Former	Current	p-value*
**Cigar**				
N	5630	251	50	
Cigar-years	0 (0)	19 (30)	47 (104)	
Glucose, mg/dL	91.9±20.2	97.2±21.9	93.8±12.8	<0.001
Insulin, mU/L	8.0 (5.7)	8.9 (6.9)	8.8 (4.1)	0.046
HOMA-IR, %	1.8 (1.5)	2.1 (1.8)	2.0 (1.2)	0.01
**Pipe**				
N	5466	435	30	
Pipe-years	0 (0)	15 (42)	100 (156)	
Glucose, mg/dL	92.0±20.3	93.9±19.5	93.6±16.8	0.001
Insulin, mU/L	8.0 (5.7)	8.5 (6.5)	10.1 (8.4)	0.03
HOMA-IR, %	1.8 (1.5)	2.0 (1.6)	2.1 (2.2)	0.01
**Smokeless**				
N	5834	70	27	
Smokeless-years	0 (0)	15 (62)	114 (164)	
Glucose, mg/dL	92.1±20.3	95.8±17.6	95.9±9.1	<0.001
Insulin, mU/L	8.0 (5.9)	8.2 (5.5)	10.7 (5.7)	0.260
HOMA-IR, %	1.8 (1.5)	1.8 (1.5)	2.6 (1.4)	0.10

Analyses are restricted to those not currently using cigarettes (n = 5123)

All variables are expressed as median (interquartile range) except for glucose which is expressed as mean ± standard deviation

* represents p-value for trend

**Table 3:** Adjusted^§^ levels of IR biomarkers by tobacco exposure status.

	Never	Former	Current	p-value**
**Cigarette**				
Glucose, mg/dL	91.4 ± 5.6	92.1 ± 6.1	91.0 ± 5.6	0.25
Insulin, mU/L	8.4 (3.1)	8.4 (3.1)	8.3 (3.2)	0.50
HOMA-IR, %	1.9 (0.8)	1.9 (0.8)	1.9 (0.8)	0.66
Cigar*				
Glucose, mg/dL	91.6 ± 5.8	94.1 ± 4.2	93.7 ± 4.7	**<0.001**
Insulin, mU/L	8.4 (3.1)	8.6 (2.6)	8.3 (2.5)	0.19
HOMA-IR, %	1.9 (0.8)	2.0 (0.7)	1.9 (0.7)	**0.03**
Pipe*				
Glucose, mg/dL	91.5 ± 5.8	93.5 ± 4.5	94.1 ± 4.7	**<0.001**
Insulin, mU/L	8.4 (3.1)	8.3 (2.7)	8.9 (1.8)	0.68
HOMA-IR, %	1.9 (0.8)	1.9 (0.7)	2.1 (0.5)	0.10
Smokeless*				
Glucose, mg/dL	91.7 ± 5.7	93.5 ± 5.5	93.5 ± 3.7	**0.004**
Insulin, mU/L	8.4 (3.1)	8.8 (3.1)	9.9 (3.5)	**0.005**
HOMA-IR, %	1.9 (0.8)	2.0 (0.8)	2.2 (0.9)	**0.003**

^§^ All adjusted values were obtained following post-estimation of linear regression models for glucose and median regression for insulin and HOMA-IR. Models were adjusted for age, sex, race/ethnicity, BMI, income, SBP, DBP, anti-hypertensive mediation, HDL, LDL, lipid-lowering medication, and alcohol use.

All variables are expressed as median (interquartile range) except for glucose, which is expressed as mean ± standard deviation

* Analysis restricted to non-current cigarette users

** Represents p-value for trend

Bolded items are significant

### IR, Dose and Intensity of Tobacco Exposure

There was a significant association between dose of tobacco exposure and levels of IR biomarkers in unadjusted or partially adjusted models, but not in fully adjusted models (Table 4). Similarly, there was no significant association between intensity of tobacco exposure and levels of IR biomarkers in fully adjusted models (Table 4). There was no association between urinary cotinine levels and levels of IR biomarkers (Table 5).

**Table 4:** Beta coefficients (95% CI) for the cross-sectional association of dose and intensity of tobacco exposure and levels of insulin resistance biomarkers.

	Dose of tobacco exposure			Intensity of tobacco exposure		
	Unadjusted	Model 1	Model 2	Unadjusted	Model 1	Model 2
**Cigarette**						
Glucose, mg/dL	**3.49 (1.01,5.97)**	**2.60 (0.10,5.11)**	1.93 (-0.43,4.29)	3,52 (-0.26,7.31)	3.75 (-0.11,7.61)	2.52 (-1.10,6.14)
Insulin, mU/L	0.03 (-0.03,0.10)	**0.08 (0.01,0.14)**	0.02 (-0.03,0.07)	0.05 (-0.0,0.15)	**0.12 (0.02,0.22)**	0.005 (-0.079,0.084)
HOMA-IR, %	0.07 (-0.04,0.14)	**0.10 (0.03,0.17)**	0.04 (-0.02,0.10)	0.09 (-0.02,0.20)	**0.16 (0.05,0.27)**	0.02 (-0.07,0.12)
**Cigar**						
Glucose, mg/dL	**2.78 (0.06,5.50)**	1.25 (-1.44,3.95)	1.08 (-1.43,3.60)	**99.96 (30.32,169.59)**	56.04 (-13.62,125.70)	34.67 (-30.84,100.17)
Insulin, mU/L	0.04 (-0.03,0.11)	0.06 (-0.01,0.13)	-0.10 (—0.03,0.08)	**2.43 (0.68,4.17)**	**3.21 (1.44,4.96)**	1.31 (-0.15,2.80)
HOMA-IR, %	**0.07 (0.01,0.15)**	0.076 (-0.002,0.153)	0.04 (-0.03,0.10)	**3.48 (1.48,5.48)**	**3.82 (1.81,5.83)**	**1.69 (0.02,3.36)**
**Pipe**						
Glucose, mg/dL	0.06 (-1.40,2.58)	-0.44 (-2.43,1.55)	-0.41 (-2.36,1.54)	3.43 (-14.77,8.33)	1.08 (-48.93,51.10)	-11.02 (-58.64,36.59)
Insulin, mU/L	**0.03 (0.02,0.08)**	**0.06 (0.01,0.11)**	0.04 (-0.01,0.08)	**1.59 (0.36,2.82)**	**2.60 (1.33,3.86)**	**1.060 (0.006,2.212)**
HOMA-IR, %	0.04 (-0.02,0.10)	**0.059 (0.002,0.117)**	0.03 (-0.02,0.08)	**1.98 (0.57,3.38)**	**2.66 (1.22,4.10)**	0.96 (-0.25,2.18)
**Smokeless tobacco**						
Glucose, mg/dL	1.15 (-3.69,5.99)	1.22 (-3.54,5.97)	1.13 (-3.63,5.89)	9.81 (-66.55,86.16)	17.33 (-57.59,92.25)	14.97(-57.44,87.39)
Insulin, mU/L	-0.01 (-0.13,0.11)	0.002 (-0.118,0.122)	-0.05 (-0.15,0.06)	-0.33 (-2.24,1.58)	-0.19 (-2.07,1.70)	-0.26 (-1.88,1.36)
HOMA-IR, %	0.01 (-0.13,0.15)	0.02 (-0.12,0.16)	-0.03 (-0.15,0.09)	-0.15 (-2.34,2.04)	0.08 (-2.08,2.22)	-0.02 (-1.86,1.82)

Glucose is untransformed while insulin and HOMA-IR are log-transformed

Dose of cigarettes is calculated as pack-years (number of cigarette packs per day multiplied by number of years), while it is calculated as cigar-years, pipe-years, and smokeless-years for cigars, pipes and smokeless tobacco respectively–(number used per day multiplied by number of years). Both are expressed per 100 years

Intensity of tobacco exposure is expressed as number of cigarette or non-cigarette tobacco used per 100 days

Model 1: age, gender, race/ethnicity

Model 2: Model 1+ BMI, income, SBP, DBP, anti-hypertensive medication, HDL, LDL, lipid-lowering medication, alcohol use

Bolded items are significant

**Table 5:** Beta coefficients (95% CI) for the cross-sectional association of cotinine levels (per 10^5 ng/mL) and measures of insulin resistance biomarkers.

	Unadjusted	Model 1	Model 2
**Glucose (mg/dL)**	-6.97 (-31.92,17.98)	-5.78 (-30.69,19.13)	5.54 (-18.18,29.26)
**Insulin(mU/L)**	-0.68 (-1.34,-0.01)	-0.87 (-1.54,-0.19)	-0.45 (-1.03,0.14)
**HOMA-IR (%)**	-0.762 (-1.520,-0.008)	-0.93 (-1.69,-0.17)	-0.40 (-1.05,0.26)

Glucose is untransformed while insulin and HOMA-IR are log-transformed

Model 1 was adjusted for age, sex, race/ethnicity

Model 2 was adjusted for Model 1 + BMI, income, SBP, DBP, anti-hypertensive medication, HDL, LDL, lipid-lowering medication, alcohol use

### Incident Type 2 Diabetes and Tobacco Exposure

There was no association between tobacco exposure and baseline pre-diabetes (Table 6). In unadjusted models, former tobacco users had a significantly increased risk of incident diabetes over a median follow-up of 10.2 years compared with never smokers [Hazard Ratio (95% confidence interval) = 1.35 (1.08,1.68), 1.74 (1.13,2.69), 1.46 (1.02,2.10) and 3.38 (1.85,6.16) for cigarette, cigar, pipe, and smokeless tobacco users respectively]. However, no association was seen between tobacco use and incident diabetes after adjustment for age, gender, race/ethnicity, BMI, income, SBP, DBP, anti-hypertensive medication, HDL, LDL, lipid-lowering medication, physical activity, healthy diet, alcohol use (Table 7). Absence of significant findings made analysis of mediation by IR unwarranted.

**Table 6:** Odds Ratios (95%) for the association of tobacco exposure and baseline prediabetes.

	Unadjusted	Model 1	Model 2
**Cigarette**			
Never	1 (reference)	1 (reference)	1 (reference)
Former	1.05 (0.90,1.22)	0.90 (0.75,1.07)	0.88 (0.72,1.08)
Current	0.91 (0.73,1.14)	0.86 (0.67,1.11)	1.02 (0.76,1.36)
**Cigar**			
Never	1 (reference)	1 (reference)	1 (reference)
Former	**1.54 (1.11,2.14)**	**1.51 (1.02,2.21)**	1.30 (0.86,1.98)
Current	1.88 (0.88,4.00)	2.16 (0.93,5.01)	1.91 (0.76,4.77)
**Pipe**			
Never	1 (reference)	1 (reference)	1 (reference)
Former	1.10 (0.83,1.46)	1.05 (0.76,1.46)	0.91 (0.63,1.32)
Current	1.85 (0.73,4.67)	2.05 (0.71,5.91)	2.04 (0.62,6.70)
**Smokeless**			
Never	1 (reference)	1 (reference)	1 (reference)
Former	1.47 (0.77,2.79)	1.28 (0.59,2.77)	0.92 (0.35,2.45)
Current	1.84 (0.50,6.80)	1.41 (0.31,6.47)	0.50 (0.04,5.66)

Prediabetes for this study was defined according to the American Diabetes Association as fasting glucose ≥100 & <126 mg/dL

Model 1 was adjusted for age, sex, race/ethnicity

Model 2 was adjusted for Model 1 + BMI, income, SBP, DBP, anti-hypertensive medication, HDL, LDL, lipid-lowering medication, physical activity, healthy diet, and alcohol use

Bolded items are significant

**Table 7:** Hazard ratios (95% CI) for the association of tobacco exposure and incident diabetes.

	N events	Unadjusted	Model 1	Model 2
**Cigarette**				
Never	154	1 (reference)	1 (reference)	1 (reference)
Former	160	1.35 (1.08,1.68)	1.20 (0.95,1.51)	1.02 (0.77,1.37)
Current	45	1.07 (0.77,1.49)	1.04 (0.74,1.46)	0.86 (0.55,1.34)
**Cigar**				
Never	331	1 (reference)	1 (reference)	1 (reference)
Former	24	1.74 (1.13,2.69)	1.51 (0.96,2.39)	1.58 (0.85,2.94)
Current	4	0.92 (0.23,3.68)	0.89 (0.22,3.61)	1.90 (0.39,9.11)
**Pipe**				
Never	321	1 (reference)	1 (reference)	1 (reference)
Former	36	1.46 (1.02,2.10)	1.43 (0.97,2.12)	1.36 (0.83,2.25)
Current	2	0.69 (0.10,4.91)	0.63 (0.09,4.49)	1.10 (0.14,8.88)
**Smokeless tobacco**				
Never	343	1 (reference)	1 (reference)	1 (reference)
Former	12	3.38 (1.85,6.16)	3.18 (1.72,5.86)	2.19 (0.83,5.76)
Current	4	3.09 (0.77,12.41)	2.75 (0.68,11.10)	−

Model 1 was adjusted for age, sex, race/ethnicity

Model 2 was adjusted for Model 1 + BMI, income, SBP, DBP, anti-hypertensive medication, HDL, LDL, lipid-lowering medication, physical activity, healthy diet, and alcohol use

### Interactions with Race, Age, and Gender

In an exploratory analysis, several outcomes, including glucose, insulin, HOMA-IR, and incident diabetes, or exposures, including cigarettes, cigars, pipe, and smokeless tobacco, were tested for interactions with race/ethnicity, age (<60 vs. >60 years of age) and gender. There were several p-values significant in the cigar, pipe and smokeless tobacco groups, including a relationship between cigarettes, race, and HOMA-IR (Table 8). However, given the small sample size of non-cigarette users and the multiple number of comparisons performed these findings may be secondary to chance alone.

**Table 8:** Interaction of race/ethnicity, age, and sex with cross-sectional insulin resistance and incident diabetes mellitus.

	Race				Age				Gender			
	Glucose	Insulin	HOMA-IR	Incident diabetes	Glucose	Insulin	HOMA-IR	Incident diabetes	Glucose	Insulin	HOMA-IR	Incident diabetes
**Cigarettes**	0.21	0.09	**0.02**	0.49	0.61	0.88	0.68	0.37	0.09	0.47	0.25	0.54
**Cigars**	0.64	**0.04**	0.06	0.15	0.67	0.65	0.51	0.90	0.47	0.19	0.15	0.70
**Pipe**	0.40	0.28	0.46	0.57	0.94	0.29	0.42	0.62	0.69	0.25	0.23	**<0.001**
**Smokeless**	0.95	0.91	0.92	0.64	0.34	0.26	0.17	0.45	0.46	0.95	0.76	**0.02**

P-value for interaction of section type (i.e. race, age, or gender) with the outcome.

Bolded items are significant

### Sensitivity analysis

In longitudinal data analyses, there was no association between dose or intensity of cigarette exposure and change in serum glucose over the course of MESA follow-up. Similarly, there was no association between dose or intensity of cigarette exposure and change in glucose from visit 1 to visit 5. There was no significant association between sustained quitters vs. sustained smokers and change in glucose levels based on dose or intensity of cigarette exposure (S1 Data). In addition, including time-varying terms for change in smoking status over time did not alter the null association between cigarette smoking status and incident diabetes (results not shown).

## Discussion

Tobacco has been shown to be associated with both subclinical markers of atherosclerosis (hsCRP, interleukin 6, fibrinogen, carotid intima-media thickness, coronary artery calcification, and ankle brachial index) [35] and the incident cardiovascular events in MESA [36]. Given these findings, we sought to examine the relationship between tobacco exposure, IR and incident diabetes as a potential mediator of tobacco induced atherosclerosis and cardiovascular events in MESA. We found no consistent association between tobacco use (cigarettes, cigars, pipe, and smokeless tobacco), and multiple measures of IR (fasting glucose, insulin, HOMA-IR) or incident diabetes regardless of whether the participants were sustained quitters or sustained smokers over 10 years of follow-up in this contemporary multiethnic cohort free of diabetes at baseline. These findings are consistent with several prior studies [22, 37, 38], but discordant with other studies, including the Surgeons General’s report, that showed a positive association between tobacco use and IR [10, 11, 39] and reports which established tobacco as a potential cause of diabetes [5–7]. We believe the discordant results in our study may be related to several factors, including: 1) changing composition of tobacco and modes of exposure through the decades, 2) differences between subjects studied, 3) inadequate adjustment for confounding, and 4) publication bias.

Temporal trends may partly be responsible for the different results seen in our study and those that report an association between tobacco use and incident DM. Indeed about half of all the studies in the Surgeon General’s report began over a quarter century ago and eight of studies examined were from individuals studied in the 1950’s through 1970’s [5], several decades before our modern cohort. Changing composition of cigarettes including the introduction of filters on cigarettes in the mid to late 1950’s which did not gain popularity until the 1980’s [18] and introduction of low tar cigarettes in the 1980’s [19] may help explain the disparate results between our study and other older cohorts. Moreover, curing of tobacco and flavoring of tobacco affect the amount and type of HPHC in the product [20, 21], which can lead to varying types of exposure to these HPHC’s.

Our study population differs from many of the epidemiological studies used in the meta-analysis, which used single race cohorts (mostly Caucasian in the Surgeon General and Willi et al. and mostly Asian in the Pan et. al. study) or did not report ethnicity [5–7]. This may lead to some of the disparate results since tobacco exposure patterns and metabolism of tobacco differs by race [20, 21, 27, 28], and gender [25, 28, 31]. Differences in smoking patterns include low rates of smoking among Hispanic/Latinos [16, 40] and greater use of cigarettes that contain menthol in African Americans [17]. Furthermore IR and diabetes rates differ by race [40, 41], gender [42], and BMI [43, 44]. MESA is a multi-ethnic, gender balanced cohort fully characterized for known risk factors of IR and DM, allowing for a uniquely complete evaluation of the association between tobacco use and IR/DM while controlling for confounders.

Unmeasured confounders in the association between IR, incident diabetes and tobacco exposure may also explain the discrepancy between studies. In our study we were able to adjust for many potential confounders (age, gender, ethnicity, BMI, income, SBP, DBP, anti-hypertensive medication, HDL, LDL, lipid-lowering medication, alcohol use). The studies included in the Surgeon General’s meta-analysis demonstrated significant heterogeneity in the number of confounders which ranged from <3 to >8. While stratified analysis of studies used in the Surgeon General’s report based on the number of these confounders did not yield substantial differences in the relationship between tobacco use and incident diabetes, many confounders were not accounted for in any of the studies used in the meta-analysis [5].

Publication bias follows from the fact that “positive” studies may be as much as three times more likely to be published than negative studies [45]. In fact, data suggests that magnitude and direction of a study's results may be a more important determinant of publication than study design, relevance, or quality [46, 47]. Publication bias is thought to be particularly prevalent for observational studies [48] and may be one contributing factor for the disparate results seen in our study and the conclusions drawn from a meta-analysis by the Surgeon General’s report.

Our analysis was performed in subjects with no known IR or diabetes at baseline in order to study the association between baseline tobacco exposure and incident type 2 diabetes. This is distinct from studies of the impact of tobacco use on IR among known type 2 diabetics or individuals with impaired glucose tolerance at the start of the study where smoking increased the magnitude of insulin resistance or glucose dysregulation [22, 24, 49, 50].

The strengths of our study include use of a well-characterized modern multi-ethnic US-based cohort with over 10 years of follow-up for incident diabetes. Cotinine measurements allowed us to reclassify tobacco use status and minimize self-report bias. Detailed information on tobacco use enabled us to evaluate for a dose response with respect to IR. Furthermore we were able to evaluate multiple forms of tobacco exposure, not just cigarettes, in relationship to IR and incident type 2 diabetes.

### Limitations

Limitations to this study include how former smokers were defined as not having used tobacco in the last 30 days. There is evidence of reversal of some of the adverse effects of tobacco may not be seen at 30 days [51]. The small sample size of non-cigarette tobacco users may not be sufficiently powered to study the association of IR and incident type 2 diabetes in these users. We acknowledge that some findings with this population may have been due to chance and therefore would benefit from further research. Furthermore, there may be a bias in our smoking cohort as they are older than some of the other populations studied. Since they are older, this cohort may have inadvertently selected for a population of smoking that had made it to older age since they were healthier than other smokers. However, we were not able to find any meaningful interactions with age in our study. In our study our smoking group had a similar or slightly higher BMI and weight compared to non and former smokers (results not shown). Previous studies have shown that smokers tend to have a lower overall BMI [11], though often a higher waist to hip ratio indicating central adiposity [52]. As BMI has been associated with IR and incident diabetes it was important to control for this covariate in our modeling. Adding this covariate separately (modeling not shown) did not change our results. Finally, we only have single cotinine measures in a subset of subjects. Since current cigarette users at the time of enrollment had different patterns of tobacco exposure (time since last cigarette), there would be considerable random error in cotinine levels especially since cotinine may only remain elevated for 18–20 hours after smoking [53].

In conclusion we found no consistent association between tobacco exposures and both cross-sectional IR and incident type 2 diabetes. Given the discordance of results with respect to tobacco use and IR / incident diabetes and the importance of type 2 diabetes to global health we believe this issue warrants further study.

## Supporting Information

S1 Data Supplementary analyses_MESA IR. (DOCX)
