# Supplementary material for: Tobacco Use, Insulin Resistance, and Risk of Type 2 Diabetes: Results from the Multi-Ethnic Study of Atherosclerosis
Source: PLoS One. 2016 Jun 20;11(6):e0157592. doi: 10.1371/journal.pone.0157592 (PMC4913922; doi:10.1371/journal.pone.0157592)
Supplement: S1 Data — (DOCX) [file pone.0157592.s001.docx]

**S1 Table.** Odds Ratios (95% confidence interval) for the association of tobacco exposure and baseline prediabetes

|  | **Unadjusted** | **Model 1** | **Model 2** |
| --- | --- | --- | --- |
| **Cigarette** |  |  |  |
| Never | 1 (reference) | 1 (reference) | 1 (reference) |
| Former | 1.05 (0.90,1.22) | 0.90 (0.75,1.07) | 0.88 (0.72,1.08) |
| Current | 0.91 (0.73,1.14) | 0.86 (0.67,1.11) | 1.02 (0.76,1.36) |
| **Cigar** |  |  |  |
| Never | 1 (reference) | 1 (reference) | 1 (reference) |
| Former | **1.54 (1.11,2.14)** | **1.51 (1.02,2.21)** | 1.30 (0.86,1.98) |
| Current | 1.88 (0.88,4.00) | 2.16 (0.93,5.01) | 1.91 (0.76,4.77) |
| **Pipe** |  |  |  |
| Never | 1 (reference) | 1 (reference) | 1 (reference) |
| Former | 1.10 (0.83,1.46) | 1.05 (0.76,1.46) | 0.91 (0.63,1.32) |
| Current | 1.85 (0.73,4.67) | 2.05 (0.71,5.91) | 2.04 (0.62,6.70) |
| **Smokeless** |  |  |  |
| Never | 1 (reference) | 1 (reference) | 1 (reference) |
| Former | 1.47 (0.77,2.79) | 1.28 (0.59,2.77) | 0.92 (0.35,2.45) |
| Current | 1.84 (0.50,6.80) | 1.41 (0.31,6.47) | 0.50 (0.04,5.66) |

Prediabetes is defined according to the American Diabetes Association as fasting glucose ≥100 & <126 mg/dL

Model 1: age, gender, race/ethnicity

Model 2: Model 1+ BMI, income, SBP, DBP, anti-hypertensive medication, HDL, LDL, lipid-lowering medication, physical activity, healthy diet, alcohol use

Bolded items are significant
